# Clock Proteins Have the Potential to Improve Term Delivery Date Prediction: A Proof-of-Concept Study

**DOI:** 10.3390/life15020224

**Published:** 2025-02-03

**Authors:** Max T. Dufford, Tracey C. Fleischer, Laura J. Sommerville, Md. Bahadur Badsha, Ashoka D. Polpitiya, Jennifer Logan, Angela C. Fox, Sharon R. Rust, Charles B. Cox, Thomas J. Garite, J. Jay Boniface, Paul E. Kearney

**Affiliations:** Sera Prognostics, Inc., Salt Lake City, UT 84109, USAtfleischer@sera.com (T.C.F.); afox@sera.com (A.C.F.); tgarite@sera.com (T.J.G.);

**Keywords:** clock proteins, term delivery date, time to birth, prediction, gestational age, a distintegrin and metalloproteinase 12

## Abstract

Our ability to accurately predict the delivery date of term pregnancies is limited by shortcomings of modern-day clinical tools and due date estimation methods. The pregnancy clock is a series of coordinated and harmonized signals between mother, fetus, and placenta that regulate the length of gestation. Clock proteins are thought to be important mediators of these signals, yet few studies have investigated their potential utility as predictors of term delivery date. In this study, we performed a cross-sectional proteome analysis of 2648 serum samples collected between 18 and 28 weeks of gestation from mothers who delivered at term. The cohort included pregnancies both with and without complications. A total of 15 proteins of diverse functionalities were shown to have a direct association with time to birth (TTB), 11 of which have not been previously linked to gestational age. The protein A Distintegrin and Metalloproteinase 12 (ADA12) was one of the 15 proteins shown to have an association with TTB. Mothers who expressed the highest levels of ADA12 in the cohort (90th percentile) gave birth earlier than mothers who expressed the lowest levels of ADA12 (10th percentile) at a statistically significant rate (median gestational age at birth 39^0/7^ weeks vs. 39^3/7^ weeks, *p* < 0.001). Altogether, these findings suggest that ADA12, as well as potentially other clock proteins, have the potential to serve as clinical predictors of term delivery date in uncomplicated pregnancies and represent an important step towards characterizing the role(s) of clock proteins in mediating pregnancy length.

## 1. Introduction

Having the capability to predict the date of term delivery (>37 weeks gestation) with certainty is an unmet need in obstetrics. Knowing precisely when labor will begin could avoid preterm birth from the mistimed scheduling of cesarian section or labor induction [1,2,3]. It could also mitigate delivery complications by allowing women who live in rural settings or maternity care deserts adequate time to reach hospital facilities [4,5,6]. Outside of a clinical setting, better estimation of when a baby will deliver has lifestyle applications since planning for this milestone affects a long list of preparative activities for the mother, their family, friends, and employer, to name a few.

In accordance with clinical guidelines, delivery date is estimated as 280 days after the woman’s last menstrual period. However, only 5% of births occur on this estimated delivery date (EDD), and 30% of births occur more than 10 days outside the EDD [7,8]. The accuracy of the EDD can be improved by a few days if it is calculated based on the fetal crown-rump length as measured by ultrasound between 8 and 12 weeks of gestation [9]. Even in developed countries, many women either lack access to ultrasounds in the first trimester or miss this crucial timing window when ultrasounds can inform gestational age [10], showing that clinical tools other than ultrasounds are needed to improve the accuracy of term delivery date prediction.

The pregnancy clock refers to a biological phenomenon in which harmonized and chronologic signals from the fetus, fetal membrane, placenta, decidua, and myometrium modulate the length of gestation [11,12,13,14]. It includes various biomarkers that comprise an immune clock, a proteomic clock, and temporal changes to the transcriptome and metabolome that correlate to gestational age based on ultrasound [14,15,16,17,18]. Some studies have shown that various biomarker components of the pregnancy clock, including protein activators of the Janus kinase and signal transducer pathways [16], lipid and steroid hormone metabolites [17], and cell-free DNA corresponding to placental genes [18], could potentially distinguish pregnancies of normal gestational length from those delivering preterm or late term and may have the capability to identify fetal maladaptations before symptoms appear.

It is recognized that maternal, paternal, and fetal factors also influence gestational length in normal pregnancies. Maternal age > 35 years and late-term birth in previous pregnancies correlate with longer gestation [19,20]. The length of pregnancy is also influenced by the body mass index (BMI) of the mother and father [21,22,23] and by the weight of the fetus in the first trimester [18]. Additionally, conceptions that take longer than 24 h to implant or that have a rapid, rather than gradual, progesterone rise during corpus luteum rescue are also associated with a longer gestational period [22].

Despite our present-day understanding of the pregnancy clock and other factors that influence gestational length, there has been little investigation into its potential utility as a clinical tool to improve the accuracy of delivery date prediction. The goal of this discovery study was to test the hypothesis that clock proteins are associated with the time to birth (TTB) and may have the capability to serve as predictors of term delivery date.

## 2. Materials and Methods

### 2.1. Materials and Reagents

Trypsin was purchased from Promega (#V5280) (Madison, WI, USA). Custom stable isotope standards were purchased from Biosynth (Staad, Switzerland). Human 14 multiple affinity removal columns (MARS-14) were purchased from Agilent (#5190-7995) (Santa Clara, CA, USA).

### 2.2. Study Design and Participants

We retrospectively analyzed 2648 banked serum samples and associated clinical and demographic data collected for an institutional-review-board-approved 11-site study in the United States (NCT01371019), which took place from April 2011 to April 2015. Inclusion and exclusion criteria for participation in the study and sample selection are shown in Table 1.

The original study aimed to characterize proteome differences in women with asymptomatic singleton pregnancies who deliver at term (>37 weeks) versus those who experience spontaneous preterm birth [24]. Prior to sample collection, enrolled individuals provided written informed consent to participate in the study and for their samples and clinical information to be used in future studies. A list of the institutions with subjects consenting to biobanking is shown in Supplementary Table S1. The samples used in the current study included term births only and were collected from 18^0/7^ through 28^6/7^ weeks of gestation at a single time point for each participant.

### 2.3. Proteomic Analysis

Whole blood samples were processed to serum at the study sites within 2.5 h of collection. Then, 0.5 mL serum aliquots were frozen at −80 °C, shipped to Sera Prognostics, Inc. on dry ice, and stored at −80 °C until analyzed. Maternal serum samples were analyzed in Sera Prognostics’ Clinical Laboratory Improvement Amendment (CLIA)-certified and College of American Pathologists (CAP)-accredited laboratory according to a prespecified laboratory analysis plan and standard operating protocols [25,26]. Serum samples were randomized and allocated into batches that contained both study samples and quality controls and processed, as described in [25]. Briefly, after thawing samples on ice, equal volumes of serum from each subject were depleted of high-abundance proteins on MARS-14 columns run on an Agilent 1260 liquid chromatography system. Depleted serum samples were reduced, alkylated, and digested with trypsin. They were then spiked with stable isotope standard (SIS) peptides corresponding to each measured endogenous peptide, and then desalted and analyzed by coupled liquid chromatography–multiple-reaction-monitoring mass spectrometry (LC-MRM-MS) on an Agilent 1290 UHPLC system coupled to an Agilent 6490 triple quadrupole mass spectrometer. We used a multiple-reaction-monitoring (MRM) assay to measure 150 peptides from 110 proteins that were either (1) of placental origin, (2) maternal serum proteins with reported roles in pregnancy, or (3) used as quality controls. Peptides were quantified as the peak area of the endogenous peptide divided by the peak area of its corresponding SIS peptide counterpart, generating a response ratio (RR). Pooled serum from either non-pregnant or pregnant women served as quality control samples and were used to monitor batch quality throughout the depletion and LC/MS/MS process, as described in [25]. Quality control samples were not used to normalize samples.

### 2.4. Statistical Analyses

A Wilcoxon test was performed to identify proteotypic peptides with mean response ratios that changed significantly between 18 and 20 weeks and 26 and 28 weeks of gestation. The results were visualized using a volcano plot. Significant difference was defined as *p* < 0.05 and a minimum log2 fold change of +/−0.25 in relative abundance. A subset of these proteins were smoothed using a generalized additive model and plotted relative to gestational age at blood draw (GABD). Confidence intervals were included to show 95% overall population values across GABD for each model. Separately, we performed a protein association study using Mendelian randomization (MR), a statistical approach to identifying causal genes that has also been applied to proteomic data [26,27,28,29]. We applied an MR-based machine learning algorithm (MRPC) with 50 iterations of bootstrapping [27,28] to identify proteins that have an association with time to birth (TTB) among individuals delivering at term. MRPC conducts a series of statistical tests for marginal and conditional independence between pairs of nodes. If the null hypothesis (i.e., no association between two nodes) of the marginal independence test fails to reject, then the corresponding edge (i.e., association between two nodes) is removed. Otherwise, the edge is retained for subsequent conditional independence test to determine if the association persists after accounting for other nodes. We used a 10% level of significance threshold (type I error rate) for each test. Finally, we compared differences in gestational age at birth (GAB) in participants having the highest (90th percentile) and lowest (10th percentile) amounts of the placental protein a distintegrin and metalloproteinase 12 (ADA12). Prior to comparing the GAB distributions, the relative abundances of ADA12 were normalized for GABD by regressing ADA12 levels against GABD and then removing any trend. The significance of the GAB mean difference was assessed using the Welch two sample *t*-test. This analysis was limited to term pregnancies with vaginal deliveries (n = 1839). We eliminated cesarean section deliveries because those are often scheduled prior to the natural onset of labor. It is unclear how many of the vaginal deliveries in the analysis were scheduled inductions, as induction data were only captured for preterm deliveries. In addition, a subset analysis of the 1839 subjects was performed to compare GAB in the highest and lowest percentiles of ADA12 levels in complicated and uncomplicated pregnancies separately. Complicated pregnancies were defined as pregnancies with diabetes (gestational or preexisting), preeclampsia, pregnancy-induced or pre-existing hypertension, incompetent cervix, polyhydramnios, chorioamnionitis, and non-reassuring fetal status. Uncomplicated pregnancies excluded the conditions listed above. All analyses and visualizations were performed using the statistical program R, version 4.4.2 [30].

## 3. Results

### 3.1. Patient Characteristics

In this retrospective study, all 2648 banked serum samples from term pregnancies were selected based on inclusion and exclusion criteria for NCT01371019 and sample selection (Table 1). Demographics of the study population were collected at the same time as blood samples and are shown in Table 2. The population was characterized by diverse clinical and demographic characteristics and pregnancy histories. While only term pregnancies were analyzed, the diverse population and the presence of pregnancy complications, including gestational diabetes, preeclampsia, and pregnancy-induced hypertension, among others, support the generalizability of these study results.

### 3.2. Clock Protein Expression

**Figure 1:**
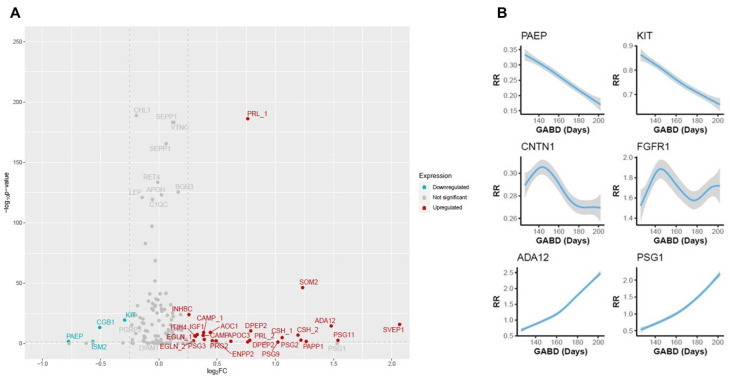
Changes in protein expression between 18 and 20 weeks of gestation and 26 to 28 weeks of gestation. (**A**) Volcano plot showing significantly upregulated (red) and downregulated (blue) proteins. Gray proteins demonstrated no significant change. Significance is represented on the *y*-axis as the −log_10_ *p*-value, while the magnitude of change is shown on the *x*-axis is the log_2_ fold-change. When a protein is depicted more than once (denoted by _1 and _2), it was measured using two distinct peptides. (**B**) Smoothing plots showing expression changes in representative proteins that were significantly upregulated (ADA12 and PSG1), downregulated (PAEP and KIT), or demonstrated no significant change (CNTN1 and FGFR1). The 95% CI between expression (response ratio (RR)) and gestational age at blood draw (GABD) is represented by the width of the gray-shaded area.

To identify proteins with levels that change during pregnancy, we first compared maternal serum protein expression at 18–20 weeks’ gestation to that at 26–28 weeks’ gestation. Changes were considered significant if protein levels satisfied both a minimum change of log_2_FC = 0.25 and achieved a *p*-value < 0.05. Several growth factors, glycoproteins, adhesion proteins, and immune regulators known to play a role in fetal growth and development demonstrated significant changes in expression (Figure 1A) that largely agree with reported findings. Both linear and non-linear expression changes were seen among samples taken between 18 and 20 weeks and 26–28 weeks of gestation. Representative changes are shown in Figure 1B, and a full listing of proteins shown in Figure 1A is provided in Supplementary Table S2.

### 3.3. Association Between Proteins and Time to Birth

**Figure 2:**
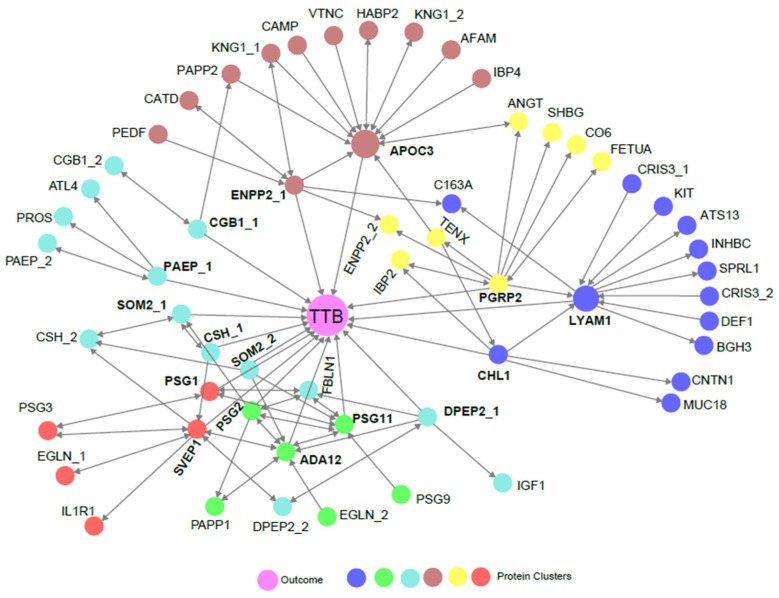
An MRPC analysis showing statistical directional relationships between proteins and TTB. Colors represent groups of proteins that are clustered based on hierarchical clustering. The outcome (TTB) is indicated by the center purple circle. Single direction arrowheads indicate statistically causal relationships where the protein or outcome being touched by the arrowhead is statistically dependent on the protein being touched by the blunt end of the arrow. Bidirectional arrows indicate indeterminant statistical causality. When a protein is depicted more than once (denoted by _1 and _2), it was measured on two distinct peptides. Proteins with a direct association to TTB are bold.

The time to birth is defined as *TTB* = (*GAB* − *GABD*), where GAB is gestational age at birth and GABD is gestational age at blood draw. To characterize associations between the proteins analyzed and TTB, we applied an MRPC [27,28] to the full protein dataset. Of the proteins analyzed (Supplementary Table S2), 15 were shown to have a direct association with TTB (Figure 2). Among these were growth hormones, adhesion molecules, glycoproteins, enzymes, and lipid-binding proteins that are involved in proliferation, migration, adhesion, fetal and placental growth, immune modulation during pregnancy, pattern recognition, and lipid metabolism (Table 3). Growth hormone 2 (SOM2), chorionic somatomammotropin (CSH), chorionic gonadotropin subunit ꞵ1 (CGB1), and sushi, von Willebrand Factor type A, EGF, and pentraxin domain-containing protein (SVEP1), are known markers of gestational age [16,31,32,33,34]. Several others, including cell adhesion molecule L1 (CHL1), lymphocyte adhesion molecule 1 (LYAM1, i.e., L-selectin), a distintegrin and metalloproteinase 12 (ADA12), ectonucleotide pyrophosphatase/phosphodiesterase 2 (ENPP2, i.e., autotaxin), apolipoprotein C-III (APOC3), and pregnancy-specific ꞵ1 glycoprotein 1 (PSG1) have been proposed as markers of certain fetal abnormalities and/or pregnancy complications [35,36,37,38,39,40,41,42,43,44,45] but have not been linked to TTB. The remaining proteins have not been previously associated with either pregnancy complications or TTB. Overall, these results identify both known and novel biomarkers that may have practical applications in predicting the probability of delivery by week in the term period.

### 3.4. Biological Association of Biomarkers with TTB

**Figure 3:**
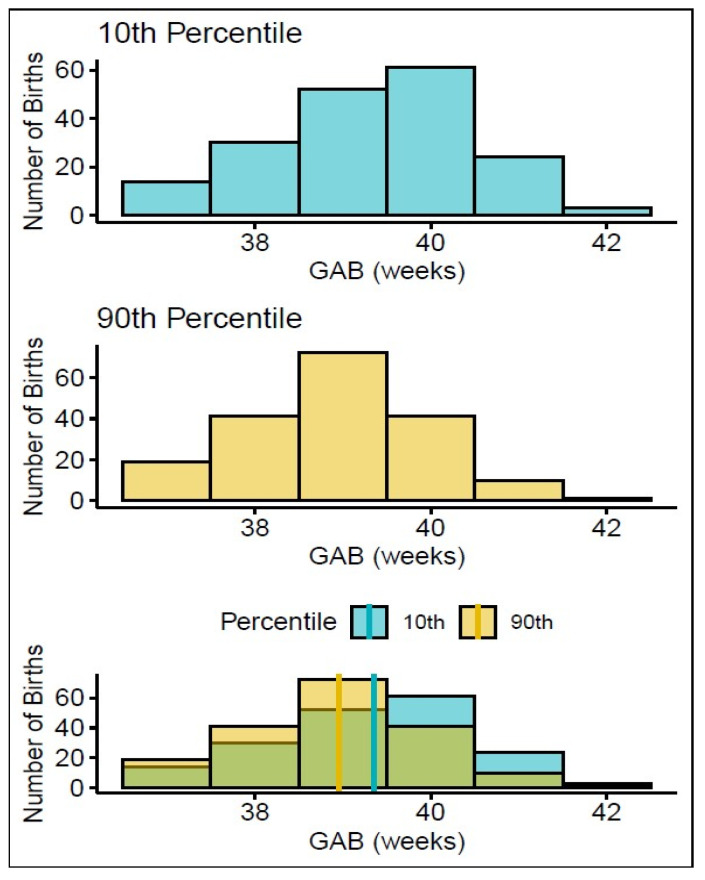
Association between GAB and ADA12 expression in all term vaginal deliveries. Graphs showing the distribution of GAB in mothers who expressed the lowest levels of ADA12 (**top**) and highest levels of ADA12 (**middle**). The combined number of births in both groups (**bottom**, green shading), shows how many more births from each group (yellow = 90th percentile, blue = 10th percentile) occurred in each week relative to the other. The solid lines indicate the mean GABs for each group: yellow = 90th percentile, blue = 10th percentile.

We next sought to visualize the impact of biomarker levels on the distribution of births. To this end, we performed a proof-of-concept analysis based on the expression of ADA12. We chose ADA12 because it had one of the largest fold changes in any protein we analyzed in this study (Figure 1A) and a near-linear association with GABD with tight confidence intervals (Figure 1B). We compared the gestational age at birth (GAB) distributions of women with the highest ADA12 levels (90th percentile) and lowest ADA12 levels (10th percentile) in all term vaginal deliveries, after normalizing for GABD, to determine if there was a difference in delivery dates. Subjects in the bottom decile of ADA12 levels had a right-skewed GAB distribution, while those in the top decile were left-skewed (Figure 3, top and middle panels). This skew was further demonstrated by significantly different mean GABs between the two groups. In the 10th percentile, the mean GAB was 39^3/7^ weeks, whereas the mean GAB in the 90th percentile was 39^0/7^ weeks, *p* < 0.001 (Figure 3, bottom panel).

To better understand how pregnancy complications might impact the predictive capability of clock proteins, we analyzed complicated and uncomplicated pregnancies separately. All complicated pregnancies, defined as those with diabetes (pre-existing or gestational), preeclampsia, pre-existing or pregnancy-induced hypertension, incompetent cervix, polyhydramnios, chorioamnionitis, or non-reassuring fetal status, were analyzed as a single group to both better power the study, and because some participants reported more than one complication. In uncomplicated pregnancies alone (n = 1242), defined as pregnancies without the above outcomes, the mean GAB of the lowest (10th percentile) ADA12 expressors was 39^5/7^ weeks compared to the mean GAB of 39^0/7^ weeks in the highest (90th percentile) ADA12 expressors. The separation of GABs in uncomplicated pregnancies alone was increased by 2 days relative to all term vaginal deliveries. In complicated pregnancies alone (n = 597), the mean GAB of both highest and lowest ADA12 expressors were both 39^0/7^ weeks. Altogether, this suggests that ADA12, and potentially other clock proteins, could aid in the prediction of TTB in uncomplicated term pregnancies.

## 4. Discussion

Having a means to accurately predict the date of term delivery could improve pregnancy outcomes by avoiding the mistimed scheduling of labor induction or cesarian section and by allowing mothers adequate time to reach hospital facilities. The pregnancy clock is understood to be a series of chronological and harmonized signals among the mother, fetus, and placenta that regulate the length of gestation. Although our understanding of the pregnancy clock proteome has advanced in recent years, the potential clinical utility of clock proteins as predictors of delivery date has not been thoroughly evaluated.

Most of the information that leads to the prediction of the timing of the normal onset of labor and delivery at term comes from dating of the mother’s last menstrual period and/or early examination and ultrasound. Often such information is unavailable, poorly remembered, or unreliable, so any surrogate means of predicting the time of delivery has great clinical and socioeconomic value. To date, most efforts to improve gestational age dating have used ultrasound, but this approach has technical limitations, and often women are unable to seek care early enough in pregnancy to provide the accurate measurements that make this tool reliable [10]. Further, our physiologic understanding of the processes leading to the timing of normal onset of labor at term is incomplete [49,50]. For these reasons, having additional means to predict when normal term labor and delivery will occur is highly important..

In this retrospective, cross-sectional study, we discovered proteins that demonstrated significant changes in expression between two gestational age windows, separated by only five weeks (Figure 1). Using MRPC analysis, we identified 15 proteins that had a direct association to the TTB, 11 of which have not been previously linked to gestational age (Figure 2). We investigated the potential clinical utility of ADA12 as a term delivery date predictor by comparing the mean and distribution of GAB between the cohort’s highest (90th percentile) and lowest (10th percentile) expressors of ADA12 at blood draw. Results showed the mean GAB to be significantly earlier in the highest ADA12 expressors compared to the lowest expressors (Figure 3). Separate analyses of complicated and uncomplicated pregnancies demonstrated an even greater separation in GAB between the highest and lowest ADA12 expressors among mothers with uncomplicated pregnancies. No separation in GAB was observed in complicated pregnancies.

Our findings on ADA12 suggest that uncomplicated pregnancies characterized by higher levels of this protein (i.e., the highest expressors in the cohort analyzed in this study) are farther advanced (shorter TTB) than those with lower levels (the lowest expressors in the study cohort). A pregnancy that is more advanced based on higher ADA12 levels and short TTB could reflect more rapid fetal development compared to pregnancies characterized by lower ADA12 levels and longer TTB. Alternatively, biomarker differences and their ability to indicate the actual TTB of pregnancy, could be a reflection of pregnancy misdating due to inaccurate recollection of when the last menstrual period occurred, limitations in fetal ultrasound measures, or lack of these measures entirely [51,52].

Regardless, based on its functional roles in pregnancy and association between its expression levels and various pregnancy complications, ADA12 may serve as an accurate predictor of term delivery date. ADA12 plays a key role in regulating trophoblast migration and invasion during early pregnancy and helps anchor trophoblast columns in the placenta during the first trimester [53]. It also cleaves insulin-like growth factor (IGF)-binding proteins that regulate the concentration of IGF, which is important for fetal growth [54]. The decreased expression of ADA12 during the first or second trimester of pregnancy has been linked to preterm birth, fetal growth restriction, preeclampsia, Down syndrome, and an increased likelihood of the fetus being small for gestational age at birth [36,41,47,55]. These observations suggest that ADA12 expression is carefully regulated in normal pregnancies and that deviation from normal levels at certain points during gestation may reflect abnormal or delayed fetal development and pregnancy complications. The direct association that we found between ADA12 and TTB and the difference in the mean GAB between the highest and lowest ADA12 expressors in our study cohort supports a role for this protein as a delivery date predictor in uncomplicated term pregnancies.

In agreement with previous studies, our findings showed a direct association between TTB and CSH, SOM2, CGB1, and SVEP1. Maternal circulating CSH increases as pregnancy progresses and correlates with STAT-5 signaling activity in CD4 T cells, an event that strongly predicts gestational age [16]. SOM2, a placentally derived hormone, is a key driver of fetal growth and placental development. It steadily increases through pregnancy, peaking around week 37 of gestation. The gestational age at peak placental SOM2 levels is associated with pregnancy length and, thus, is also an indicator of delivery date [31]. CGB1 is the subunit that gives human chorionic gonadotropin (hCG) its functional specificity. hCG is critical for fetal viability as it plays a central role in thickening the uterine lining, stimulating progesterone production, stopping menstruation, and enhancing embryo implantation and survival [56]. Quantitative measurements of hCG are currently used in clinical practice to calculate gestational age [33]. In addition, one study showed that, when the CGB transcript was included in a model that contained measurements of eight cfRNAs, it was predictive of delivery within 14 days of the actual date with an accuracy ranging from 23 to 45% depending on trimester, roughly comparable to ultrasound with an accuracy of 48% [18]. SVEP1, which is also upregulated in pregnancy, has been shown to correlate with gestational age of chorionic villi [32]. Each of these proteins is placentally derived, and placental–fetal signaling plays a crucial role in the pregnancy clock and triggering of parturition.

Many of the 15 proteins shown to have a direct association with TTB have placental expression, including pregnancy-specific ꞵ1 glycoprotein 1 (PSG1). PSG1 binds to heparin sulfate proteoglycans, the latency-associated peptide of TGF-β1, and the platelet integrin αIIꞵ3 [57,58]. Through interactions with these ligands, PSG1 helps mediate the shift away from innate immunity in pregnancy [59,60] and dampens platelet aggregation and thrombosis to counterbalance the prothrombotic maternal environment of pregnancy [58]. PSG1 expression gradually increases throughout pregnancy, reaching its peak around 36 weeks of gestation [61]. In our study, PSG1 expression increased during the 8–10-week timeframe studied but not to statistical significance. Since the smoothing plot shows a linear association with GABD and small confidence intervals, this was likely due to the gestational timeframe for blood draw being too narrow to reach significance. Other PSG proteins measured in our study had similar linear associations with GABD. Interestingly, the transcript for PSG7 was included in the 8-cfRNA model described above.

Another placental protein identified here, PAEP, helps modulate the immune system in pregnancy. During the second trimester, a tightly regulated suppression of the immune system occurs to prevent the mother from rejecting the fetus. This suppression is characterized by a shift towards a Th2 immune response, which decreases the number of several types of innate immune cells including natural killer (NK) cells [62,63]. PAEP has been shown to facilitate this natural immune shift by mediating apoptosis of NK cells [64,65]. Our findings suggest that PAEP and other proteins involved in the immune system shift may also serve as a type of signaling molecule to indicate the progress of gestation.

The abnormal expression or disrupted function of several of the other identified proteins has been associated with pregnancy complications and/or fetal abnormalities, but, to our knowledge, none of these have previously been associated with TTB. For instance, CHL1 is an adhesion molecule that is expressed on the surface of neurons and plays a role in their migration and organization. Mutations in the *CHL1* gene result in brain malformation and neurodevelopmental delay [42], and neurological phenotypes attributed to fetal alcohol syndrome have been partially attributed to disruption of functions mediated by CHL1 [37,38,66]. CHL1 is highly expressed in the developing fetal spinal cord and in extracellular vesicles, suggesting that during pregnancy it serves as an extracellular signaling molecule to support axon outgrowth. [67]. Our study suggests that CHL1-mediated activity may also serve as a clock signal that influences TTB.

LYAM1 (L-selectin) is an adhesion molecule expressed on the surface of leukocytes, blastocysts and in cytotrophoblast aggregates. This protein facilitates blastocyst adhesion to the endometrium during pregnancy to enable embryo implantation and is essential for the proper anchoring of the fetus to the decidua [68,69]. LYAM1 is highly expressed at the beginning of pregnancy, then tapers during the second trimester [70]. Its levels may remain elevated in preeclampsia, likely because of increased leukocyte activation stemming from inflammatory signaling [35]. The novel association between LYAM1 and TTB reported in this study aligns with its role as a mediator of implantation and protector of fetal placement during pregnancy.

One protein identified in our study has not been associated with normal pregnancy processes, pregnancy complications, or gestational age. PGRP2 is a pattern recognition molecule, expressed mainly in gut epithelial cells, that recognizes and hydrolyzes bacterial peptidoglycan (PGN) [71,72]. Bacterial PGN derived from the gut microbiota has been shown to influence brain development [73], and PGRP2 has been implicated as a mediator of this phenomenon, owing to its influence on expression of brain-derived neurotropic factor and the autism risk gene *c-Met* [74]. Our findings suggest a novel role for this protein as an indicator of pregnancy timing.

Although these findings are intriguing, we acknowledge this study’s limitations. Since only 110 proteins were measured in the MRM assay, it is possible that the direct associations we report between certain proteins and TTB may include protein intermediates, which could be identified in a larger analysis. This study was not based on longitudinal sampling; therefore, we could not make direct associations between changes in protein expression and outcome in the same study participant. However, since the proteins shown to be associated with TTB were identified in a large cross-sectional cohort, the associations we found are likely applicable to the general population. This study did not investigate the contribution of clinical variables to the prediction of TTB. For example, in our cohort, there was a significant difference in GAB between primigravida vs. multigravida women (276.1 vs. 274.0 days, respectively, *p* < 0.001). The cohort was not well annotated regarding reasons for labor induction/augmentation so some gestational ages at delivery may have been a result of scheduled delivery. Additionally, although medications were noted in the Case Report Form, there was not sufficient granularity to know which medications were present at the time of blood draw. The observation that the mean GABs were not different between the highest and lowest ADA12 expressors in complicated pregnancies, suggests that pregnancy complications may impact the predictive capability of ADA12 or other clock proteins. However, our ability to evaluate the impact of these variables is unfortunately limited because clinical decision-making and the response and adherence to treatment for study participants who experienced complicated pregnancies are unknown. Further investigation is needed to better characterize the relationship(s) between pregnancy complications and gestational length. In the future, any algorithm utilizing clock proteins to predict TTB may need to include the existence of certain demographic or clinical factors, such as maternal BMI or age, or require updating predictions as complications develop in the index pregnancy.

## 5. Conclusions

This study demonstrated proof-of-concept for ADA12 as a potential biomarker for predicting the delivery date of uncomplicated term pregnancy and identified a direct association between TTB and 15 proteins, 11 of which have not been previously linked to delivery date prediction or gestational age. Overall, this discovery work represents an important first step towards characterizing the relationship between clock protein behavior and the duration of pregnancy and determining their clinical utility as predictors of delivery date in term pregnancies.

## Figures and Tables

**Table 1:** Study inclusion and exclusion criteria.

Inclusion Criteria	Exclusion Criteria
Subject is 18 years or older Subject has a singleton pregnancy Subject can provide consent Subject consented to use of their specimens and data for future research	Subject is pregnant with more than one fetus There is a known or suspected fetal anomaly Early discontinued (consent errors, duplicate enrollment, unable to complete blood draw, gestational age (GA) window violation, inclusion/exclusion violations, sample handling errors, etc.) Early withdraw, lost to follow-up Insufficient sample amount

**Table 2:** Demographics of study participants.

Demographic/Clinical Variable	Value	All Subjects (n = 2648)
**Maternal Age**	Median (IQR)	28 years (23–32)
**Maternal Pre-pregnancy BMI**	Median (IQR)	25.90 (22.3–31.4) *
**Gravida**	Primigravida	710 (26.8 %)
	Multigravida	1938 (73.2 %)
**Race**	Black	414 (15.6%)
	White	1998 (75.5%)
	Other	236 (8.9%)
**Ethnicity**	Hispanic	942 (35.6%)
	Not Hispanic	1706 (64.4%)
**Gestational age at blood draw (GABD)**	Median (IQR)	166 days (141–184)
**Gestational age at birth (GAB)**	Median (IQR)	274 days (270–280)
Diabetes **	Gestational	183 (6.9%)
	Type I	19 (0.7%)
	Type II	55 (2.1%)
	None	2391 (90.3%)
**Pre-eclampsia**	No	2516 (95.0%)
	Yes	132 (5.0%)
**Pregnancy-Induced Hypertension**	No	2580 (97.4%)
	Yes	68 (2.6%)
Other Complications ***	No	2455 (92.7%)
	Yes	193 (7.3%)
**Delivery**	Cesarean Section: Primary	405 (15.3%)
	Cesarean Section: Repeat	404 (15.3%)
	Vaginal	1839 (69.4%)

* A total of 45 values for maternal pre-pregnancy BMI were missing from the dataset. ** Type I and type II diabetes indicate study participants who were diagnosed with diabetes before pregnancy. *** Other complications include incompetent cervix, polyhydramnios, chorioamnionitis, and non-reassuring fetal status.

**Table 3:** Summary of proteins shown to have a direct association with TTB. NS = not significant.

Protein (UniProt ID)	Expression Change Between 18 and 20 Weeks of Gestation and 26 and 28 Weeks of Gestation, Wilcoxon Test	Placentally Expressed [46]	Protein Type	Role in Pregnancy	Previously Reported Associations with TTB or Pregnancy Complications
Peptidoglycan Recognition Protein 2 (PGRP2)	Downregulated NS	Yes	Hydrolase enzyme	Pattern recognition protein	None
Cell Adhesion Molecule L1 Like (CHL1)	Downregulated NS	No	Cell adhesion molecule	Negative regulator of proliferation	Brain malformation and neurodevelopmental delay [37,38,42]
Lymphocyte Adhesion Molecule 1 (LYAM1)	Downregulated NS	No	Cell adhesion molecule	Cell adhesion and migration	Preeclampsia [35]
Dipeptidase 2 (DPEP2)	Upregulated	Yes	Hydrolase enzyme	Cell differentiation	None
Growth Hormone 2 (SOM2)	Upregulated	Yes	Growth hormone	Cell differentiation and proliferation	Marker of gestational age [31]
Chorionic somatomammotropin (CSH) *	Upregulated	Yes	Growth hormone	Stimulates lactation and fetal growth	Marker of gestational age [16]
Progestogen-associated endometrial protein (PAEP)	Downregulated	Yes	Glycoprotein	Regulates uterine environment/immune cell inhibitor	None
Chorionic Gonadotropin Subunit Beta 1 (CGB1)	Downregulated	Yes	ꞵ-subunit of human chorionic gonadotropin	Stimulates uterine and fetal growth/immune modulator	Marker of gestational age [33]
Pregnancy-Specific ꞵ1 Glycoprotein 11 (PSG11)	Upregulated	Yes	Placental glycoprotein	Immune cell and angiogenesis modulator	None
Disintegrin and metalloproteinase domain-containing protein 12 (ADA12)	Upregulated	Yes	Placental glycoprotein	Placental growth and differentiation	Preterm birth, fetal growth restriction, preeclampsia, Down Syndrome, small for gestational age [36,41,47]
Pregnancy-Specific ꞵ1 Glycoprotein 2 (PSG2)	Upregulated	Yes	Placental glycoprotein	Immune cell and angiogenesis modulator	None
Sushi, von Willebrand Factor type A, EGF, and pentraxin domain-containing protein (SVEP1)	Upregulated	Yes	Cell adhesion molecule	Facilitates cell alignment and migration	Marker of gestational age [32]
Pregnancy-Specific ꞵ1 Glycoprotein 1 (PSG1)	Upregulated NS	Yes	Placental glycoprotein	Immune cell and angiogenesis modulator	Preeclampsia [48]
Ectonucleotide Pyrophosphatase/Phosphodiesterase 2 (ENPP2)	Upregulated	Yes	Phosphodiesterase and phospholipase	Cell proliferation and migration	Preeclampsia [39,40]
Apolipoprotein C-III (APOC3)	Upregulated	No	Lipid binding protein	Modulator of lipid metabolism	Gestational Diabetes Mellitus [44]

* Since their protein sequences differ by only one amino acid, our methods cannot distinguish CSH1 from CSH2.

## Data Availability

Data supporting the results presented here are available upon request from the corresponding author. Data will not be made publicly available or in any format that may violate a subject’s right to privacy.

## Supplementary Materials

The following supporting information can be downloaded at: https://www.mdpi.com/article/10.3390/life15020224/s1, Table S1: Study cites that agreed to biobanking; Table S2: Proteins identified as regulated or identified as directly or indirectly associated with time to birth.

## Abbreviations

The following abbreviations are used in this manuscript:

ADA12	Disintegrin and metalloproteinase domain-containing protein 12
AFAM	Afamin
ANGT	Angiotensinogen
AOC1	Amiloride-sensitive amine oxidase [copper-containing]
APOC3	Apolipoprotein C-III
APOH	Beta-2-glycoprotein 1
ATL4	ADAMTS-like protein 4
ATS13	A disintegrin and metalloproteinase with thrombospondin motifs 13
BGH3	Transforming growth factor-beta-induced protein ig-h3
BMI	Body mass index
C163A	Scavenger receptor cysteine-rich type 1 protein M130
C1QC	Complement C1q subcomponent subunit C
CAMP	Cathelicidin antimicrobial peptide
CAP	College of American Pathologists
CATD	Cathepsin D
CGB1	Choriogonadotropin subunit beta variant 1
CHL1	Neural cell adhesion molecule L1-like protein
CLIA	Clinical Laboratory Improvement Amendment
CNTN1	Contactin-1
CO6	Complement component C6
CRIS3	Cysteine-rich secretory protein 3
CSH	Chorionic somatomammotropin hormone 1
CSH	Chorionic somatomammotropin hormone 2
DEF1	Neutrophil defensin 1
DPEP2	Dipeptidase 2
EDD	Estimated delivery date
EGLN	Endoglin
ENPP2	Ectonucleotide pyrophosphatase/phosphodiesterase family member 2
FBLN1	Fibulin-1
FETUA	Alpha-2-HS-glycoprotein
HABP2	Hyaluronan-binding protein 2
GAB	Gestational age at birth
GABD	Gestational age at blood draw
IBP2	Insulin-like growth factor-binding protein 2
IBP4	Insulin-like growth factor-binding protein 4
IGF1	Insulin-like growth factor I
IL1R	Interleukin-1 receptor type 1
INHBC	Inhibin beta C chain
ISM2	Isthmin-2
ITIH4	Inter-alpha-trypsin inhibitor heavy chain H4
KIT	Mast/stem cell growth factor receptor Kit
KNG1	Kininogen-1
LC-MRM-MS	Liquid chromatography–multiple reaction monitoring–mass spectrometry
LEP	Leptin
LYAM1	L-selectin
MARS	Multiple affinity removal system
MR	Mendelian randomization
MRPC	Mendelian randomization-based machine learning algorithm
MRM	Multiple reaction monitoring
MUC18	Cell surface glycoprotein MUC18
PAEP	Glycodelin
PAPP1	Pappalysin-1
PAPP2	Pappalysin-2
PEDF	Pigment epithelium-derived factor
PGRP2	*n*-acetylmuramoyl-L-alanine amidase
PRG2	Bone marrow proteoglycan
PRL	Prolactin
PROS	Vitamin K-dependent protein S
PSG1	Pregnancy-specific beta-1-glycoprotein 1
PSG11	Pregnancy-specific beta-1-glycoprotein 11
PSG2	Pregnancy-specific beta-1-glycoprotein 2
PSG3	Pregnancy-specific beta-1-glycoprotein 3
PSG9	Pregnancy-specific beta-1-glycoprotein 9
RET4	Retinol-binding protein 4
RR	Response ratio
SEPP1	Selenoprotein P
SHBG	Sex hormone-binding globulin
SOM2	Growth hormone variant
SPRL1	SPARC-like protein 1
SIS	Stable isotope standard
SVEP1	Sushi, von Willebrand factor type A, EGF and pentraxin domain-containing protein 1
TENX	Tenascin-X
TTB	Time to birth
UHPLC	Ultra-high-performance liquid chromatography
VTNC	Vitronectin
