# Supplementary material for: Clock Proteins Have the Potential to Improve Term Delivery Date Prediction: A Proof-of-Concept Study
Source: Life (Basel). 2025 Feb 3;15(2):224. doi: 10.3390/life15020224 (PMC11856609; doi:10.3390/life15020224)
Supplement: Supplementary file 1 [file life-15-00224-s001.zip › life-3412420-supplementary.pdf]

Supplemental Materials

Supplemental Table S1. Sample collection sites.	
Institution	Location
The University of North Carolina	Chapel Hill, North Carolina
Medical University of South Carolina	Charleston, South Carolina
Maricopia Integrated Health System	Phoenix, Arizona
Ohio State University	Columbus, Ohio
Baystate Medical Center	Springfield, Massachusetts
Oregon Health and Science University	Portland, Oregon
San Diego Perinatal Clinic	San Diego, California
University of Texas medical Branch at Galveston	Galveston, Texas
Christiana Care Health System	Cristiana, Delaware
Regional Obstetrical Consultants	Chattanooga, Tennessee

Supplemental Table S2. Proteins identified as regulated or directly or indirectly associated with time to birth.		
**Protein Name**	**Abbreviation**	**Uniprot Short Name**
Disintegrin and metalloproteinase domain-containing protein 12	ADA12	O43184
Afamin	AFAM	P43652
Angiotensinogen	ANGT	P01019
Amiloride-sensitive amine oxidase [copper-containing]	AOC1	P19801
Apolipoprotein C-III	APOC3	P02656
Beta-2-glycoprotein 1	APOH	P02749
ADAMTS-like protein 4	ATL4	Q6UY14
A disintegrin and metalloproteinase with thrombospondin motifs 13	ATS13	Q76LX8
Transforming growth factor-beta-induced protein ig-h3	BGH3	Q15582
Scavenger receptor cysteine-rich type 1 protein M130	C163A	Q86VB7
Complement C1q subcomponent subunit C	C1QC	P02747
Cathelicidin antimicrobial peptide	CAMP	P49913
Cathepsin D	CATD	P07339
Choriogonadotropin subunit beta variant 1	CGB1	A6NKQ9
Neural cell adhesion molecule L1-like protein	CHL1	O00533
Contactin-1	CNTN1	Q12860
Complement component C6	CO6	P13671
Cysteine-rich secretory protein 3	CRIS3	P54108
Chorionic somatomammotropin hormone 1	CSH	P0DML2
Chorionic somatomammotropin hormone 2	CSH	P0DML3
Neutrophil defensin 1	DEF1	P59665
Dipeptidase 2	DPEP2	Q9H4A9
Endoglin	EGLN	P17813
Ectonucleotide pyrophosphatase/phosphodiesterase family member 2	ENPP2	Q13822
Fibulin-1	FBLN1	P23142
Alpha-2-HS-glycoprotein	FETUA	P02765
Hyaluronan-binding protein 2	HABP2	Q14520

Insulin-like growth factor-binding protein 2	IBP2	P18065
Insulin-like growth factor-binding protein 4	IBP4	P22692
Insulin-like growth factor I	IGF1	P05019
Interleukin-1 receptor type 1	IL1R	P14778
Inhibin beta C chain	INHBC	P55103
Isthmin-2	ISM2	Q6H9L7
Inter-alpha-trypsin inhibitor heavy chain H4	ITIH4	Q14624
Mast/stem cell growth factor receptor Kit	KIT	P10721
Kininogen-1	KNG1	P01042
Leptin	LEP	P41159
L-selectin	LYAM1	P14151
Cell surface glycoprotein MUC18	MUC18	P43121
Glycodelin	PAEP	P09466
Pappalysin-1	PAPP1	Q13219
Pappalysin-2	PAPP2	Q9BXP8
Pigment epithelium-derived factor	PEDF	P36955
N-acetylmuramoyl-L-alanine amidase	PGRP2	Q96PD5
Bone marrow proteoglycan	PRG2	P13727
Prolactin	PRL	P01236
Vitamin K-dependent protein S	PROS	P07225
Pregnancy-specific beta-1-glycoprotein 1	PSG1	P11464
Pregnancy-specific beta-1-glycoprotein 11	PSG11	Q9UQ72
Pregnancy-specific beta-1-glycoprotein 2	PSG2	P11465
Pregnancy-specific beta-1-glycoprotein 3	PSG3	Q16557
Pregnancy-specific beta-1-glycoprotein 9	PSG9	Q00887
Retinol-binding protein 4	RET4	P02753
Selenoprotein P	SEPP1	P49908
Sex hormone-binding globulin	SHBG	P04278
Growth hormone variant	SOM2	P01242
SPARC-like protein 1	SPRL1	Q14515
Sushi, von Willebrand factor type A, EGF and pentraxin domain-containing protein 1	SVEP1	Q4LDE5
Tenascin-X	TENX	P22105
Vitronectin	VTNC	P04004
